# Utilizing the Patient Care Process to Minimize the Risk of Vancomycin-Associated Nephrotoxicity

**DOI:** 10.3390/jcm8060781

**Published:** 2019-06-01

**Authors:** Ashley R. Selby, Ronald G. Hall

**Affiliations:** 1 Department of Pharmacy Practice, Texas Tech University Health Sciences Center Jerry H. Hodge School of Pharmacy, Dallas, TX 75235, USA; Ronald.Hall@ttuhsc.edu; 2 VA North Texas Health Care System, Dallas, TX 75216, USA; 3 Department of Surgery, University of Texas Southwestern Medical Center, Dallas, TX 75390, USA; 4 Dose Optimization and Outcomes Research (DOOR) Program, Dallas, TX 75235, USA

**Keywords:** vancomycin, MRSA, nephrotoxicity, acute kidney injury, piperacillin-tazobactam, creatinine, KIM-1, AKIN, KDIGO, RIFLE

## Abstract

Vancomycin-associated acute kidney injury (AKI) is a popular topic in the medical literature with few clear answers. While many studies evaluate the risk of AKI associated with vancomycin, few data are high quality and/or long in duration of follow-up. This review takes the clinician through an approach to evaluate a patient for risk of AKI. This evaluation should include patient assessment, antibiotic prescription, duration, and monitoring. Patient assessment involves evaluating severity of illness, baseline renal function, hypotension/vasopressor use, and concomitant nephrotoxins. Evaluation of antibiotic prescription includes evaluating the need for methicillin-resistant *Staphylococcus aureus* (MRSA) coverage and/or vancomycin use. Duration of therapy has been shown to increase the risk of AKI. Efforts to de-escalate vancomycin from the antimicrobial regimen, including MRSA nasal swabs and rapid diagnostics, should be used to lessen the likelihood of AKI. Adequate monitoring includes therapeutic drug monitoring, ongoing fluid status evaluations, and a continual reassessment of AKI risk. The issues with serum creatinine make the timely evaluation of renal function and diagnosis of the cause of AKI problematic. Most notably, concomitant piperacillin-tazobactam can increase serum creatinine via tubular secretion, resulting in higher rates of AKI being reported. The few studies evaluating the long-term prognosis of AKI in patients receiving vancomycin have found that few patients require renal replacement therapy and that the long-term risk of death is unaffected for patients surviving after the initial 28-day period.

## 1. Introduction

Vancomycin has been a mainstay of empiric therapy for gram-positive pathogens, particularly methicillin-resistant *Staphylococcus aureus* (MRSA), for over 50 years. The history of vancomycin has also been littered with safety concerns since the days of “Mississippi Mud”, which the impure formulations of vancomycin were affectionately called. In the 1980s, nephrotoxicity concerns rose again. These concerns largely went away as studies found that this nephrotoxicity was generally reversible and randomized; controlled trials of one gram of vancomycin every 12 h reported nephrotoxicity rates of 0–5% [1,2,3].

Efficacy concerns prompted the development of the vancomycin consensus document. The 2009 consensus statement recommended trough concentrations of 15–20 mg/L for severe infections in an attempt to overcome increasing vancomycin minimum inhibitory concentrations (MICs) in MRSA [4]. The unintended consequence of this recommendation was a significant increase in the rate of nephrotoxicity reported in the literature. However, it is unclear how much of this increase was due to increased trough concentrations versus the more stringent nephrotoxicity definitions that were being adopted into routine use for research.

Vancomycin use rose by 32% from 2006 to 2012 in the US despite increasing fears regarding nephrotoxicity [5]. Therefore, many clinicians still have faith in vancomycin as a relatively safe antimicrobial despite multiple observational reports and one randomized, controlled trial suggesting otherwise [1,4,5].

The discordance between the data associating vancomycin with nephrotoxicity (including unclear dosing and monitoring requirements) and routine antibiotic prescribing patterns for MRSA infections leave the reasonable clinician debating the best course of action regarding how to incorporate this literature into practice. This review aims to walk the reader through the patient care process (Table 1), analyzing potential factors associated with the development of vancomycin-associated nephrotoxicity or its outcomes during each step.

## 2. Patient Assessment

Every patient that receives vancomycin is not the same. The baseline risk of nephrotoxicity varies based on several factors. These factors include the patient’s baseline severity of illness, concomitant disease states, and concomitant nephrotoxins. This means that the patients are likely to have a higher or lower baseline risk of nephrotoxicity based on the presence or absence of the factors that will be discussed in this section.

Several patient characteristics can be utilized to indicate a patient’s severity of illness. These variables are not routinely evaluated together in a multivariable model due to concerns regarding collinearity. Multiple studies have found that the risk of nephrotoxicity increases as the baseline APACHE II score increases [6,7]. We have also found that an increased Pitt bacteremia score was associated with nephrotoxicity in patients with MRSA bacteremia [8]. The impact of increasing Sequential Organ Failure Assessmentscores on nephrotoxicity has not been studied, to our knowledge. Intensive care unit residence has also been associated with vancomycin-associated nephrotoxicity in two retrospective studies by Lodise and colleagues [9,10].

Renal dysfunction at baseline has also been associated with nephrotoxicity in multiple studies. Baseline serum creatinine levels ≥1.7 or 2.0 mg/dL were found to be independent predictors of nephrotoxicity in retrospective analyses [11,12]. We have also found that evaluating baseline serum creatinine as a continuous variable (1 mg/dL increments) is also associated with nephrotoxicity [13]. A computer-guided cutpoint of an estimated CrCl ≤ 86.6 mL/min was also associated with time to nephrotoxicity (adjusted odds ratio (OR) 3.7; 95% confidence interval (CI) 1.2–11.5) [9]. The mechanism of why impaired renal function would play a role in the development of nephrotoxicity has yet to be fully elucidated. Some potential reasons would include increased drug exposure through decreased baseline renal function as well the pre-existing kidney damage noted by an increased serum creatinine (and possible diagnosis of chronic kidney disease). The finding by Rutter and colleagues of increased creatinine clearance being associated with nephrotoxicity further adds to the uncertainty regarding this potential factor [14].

Several studies have also reported the association between vasopressor use and nephrotoxicity. These studies do not report information regarding the duration of hypotension prior to vasopressor use [6,12,15,16]. This means that vasopressor use is sometimes used as a surrogate marker of hypotension. It is unknown whether the hypotensive episode or vasopressor use has a greater impact on the development of nephrotoxicity. The majority of studies that have evaluated the impact of hypotensive events on nephrotoxicity have had limited numbers of patients having hypotensive events [17,18,19]. Rutter and colleagues found hypotensive events to be significantly associated with nephrotoxicity in the largest study to evaluate the impact of hypotensive events in patients receiving vancomycin [14].

Receipt of other nephrotoxic agents may also contribute to nephrotoxicity. A systematic review demonstrated that patients receiving concomitant nephrotoxins were more likely to develop nephrotoxicity (OR 3.30; 95% CI 1.30–8.39) [20]. The role of individual agents, including dose and/or duration, is more difficult to ascertain given that most currently available data have very few events and only allow for the evaluation of a few select covariates. Models that attempt to evaluate too many variables compared to the number of events in the study suffer from overfitting issues, compromising their external validity.

Nephrotoxicity is a known risk associated with the use of aminoglycosides and amphotericin B [21,22]. The use of concomitant aminoglycoside or amphotericin B in addition to vancomycin was the only factor independently associated with nephrotoxicity in one multivariate analysis [23]. Aminoglycosides were also the only concomitant medication associated with nephrotoxicity in a study specifically evaluating critically ill patients [24].

Two large, retrospective cohort studies of hospitalized patients suggest that nephrotoxicity is associated with concomitant angiotensin converting enzyme inhibitor, amphotericin B, tacrolimus, loop diuretics, and tenofovir [14,25]. The concomitant use of a loop diuretic in patients receiving vancomycin and an antipseudomonal beta-lactam was associated with nephrotoxicity in a multicenter observational study (OR 3.27; 95% CI 1.42–7.53) [16].

The concomitant receipt of piperacillin-tazobactam has been the focus of most recent studies regarding vancomycin-associated nephrotoxicity. Several studies have highlighted the increased risk of acute kidney injury (AKI) associated with concomitant receipt of piperacillin-tazobactam with vancomycin [16,26,27,28]. Some studies focused on patients admitted to the intensive care unit have not found this association [29,30]. Schreier and colleagues demonstrated that the empiric use of this combination is not associated with nephrotoxicity when de-escalation occurs within the first 48–72 h [31].

The mechanisms for the increased rates of nephrotoxicity with piperacillin-tazobactam have been unclear. Data suggest that the association is not due to the beta-lactamase inhibitor or the infusion strategy [32]. Some have even suggested that the increase in serum creatinine with piperacillin-tazobactam does not represent nephrotoxicity in these patients. Piperacillin-tazobactam is known to increase serum creatinine through inhibition of creatinine tubular secretion without decreasing glomerular filtration rate [33]. There are also clinical data suggesting that the addition of piperacillin-tazobactam to vancomycin lowers dialysis rates even in the face of increased rates of AKI as measured by increases in serum creatinine [29]. Data from a benchtop animal study suggest that the concomitant use of piperacillin/tazobactam may delay the increase in kidney injury molecule-1 (KIM-1) in animals receiving vancomycin [34]. Therefore, piperacillin-tazobactam may be renal-protective even though it increases serum creatinine.

## 3. Antibiotic Prescription

Up to 50% of inpatient antimicrobial use has been shown to be inappropriate [35]. A recent study has also shown that vancomycin remains one of the most commonly used antimicrobials in hospitals [5]. This is in part due to the high prevalence of methicillin-resistance amongst *S. aureus* isolates as well as the pressures to ensure adequate empiric coverage for the suspected infection. Adding to the concern are diagnostic dilemmas including inconclusive radiographic evidence of infection and the era of health-care associated pneumonia that dramatically increased vancomycin use. A patient-by-patient assessment MRSA risk is needed to avoid the overprescribing of empiric MRSA coverage, which will hopefully be aided in the future by better risk scores and/or rapid diagnostics beyond nasal swabs.

Given the high prevalence of methicillin-resistance amongst *S. aureus* isolates, empiric therapy with vancomycin is common. This is in part due to its inclusion as a first-line option for MRSA in Infectious Diseases Society of America (IDSA) guidelines for skin and soft-tissue infections, diabetic foot infections, endocarditis, febrile neutropenia, meningitis, pneumonia, and surgical prophylaxis [36,37,38,39,40,41,42,43].

However, there are clinical scenarios where vancomycin is not the optimal agent for definitive therapy. There are currently seven oral and 11 intravenous agents that are approved by the U.S. Food and Drug Administration that are active against MRSA. Vancomycin should be evaluated against these other options to determine the optimal agent for a particular patient. Vancomycin is not the optimal agent for a patient that is eligible for oral antimicrobial therapy, as multiple studies have shown the non-inferiority of oral antimicrobials for serious infections [44,45].

## 4. Duration of Therapy

Several studies have demonstrated that the risk of nephrotoxicity is associated with the duration of vancomycin therapy [27]. Multiple studies have shown that the risk of nephrotoxicity increases after four days of therapy [9,10,15,19,20]. Others have found that a duration of therapy of seven or 14–15 days is associated with nephrotoxicity [6,8,11]. Another study found that the rates of nephrotoxicity increased when the duration was extended from seven or fewer days (6%) to 8–14 days (21%), and to 30% when extended >14 days [23]. Most patients should not require vancomycin for more than seven days [36,39,41,42,43]. Some notable exceptions include osteomyelitis and endocarditis [37,38].

De-escalation is a sound antimicrobial strategy for several reasons, including reducing vancomycin duration and possibly the risk of nephrotoxicity. A retrospective study found that the de-escalation of anti-MRSA agents in culture-negative nosocomial pneumonia within the first four days of empiric therapy was associated with a lower rate of AKI (36% vs. 50%; difference, −13.8%; 95% CI −26.9 to −0.4) [46]. Rapid diagnostic tests may further assist with de-escalation due to their strong negative predictive value [47,48]. The use of MRSA polymerase chain reaction (PCR) testing shortened the duration of anti-MRSA coverage in a small retrospective study by approximately two days and was associated with decreased rates of AKI (26% vs. 3%; *p* = 0.02) [49].

## 5. Monitoring

### 5.1. Vancomycin Concentrations

The IDSA/Society for Healthcare Epidemiology of America (SHEA) antimicrobial stewardship guidelines provide a weak recommendation for the therapeutic drug monitoring of vancomycin based on low-quality evidence [50]. To date, only one randomized controlled trial has evaluated the impact of vancomycin therapeutic drug monitoring on the development of nephrotoxicity (serum creatinine increase of 0.5 mg/dL or more) [51]. This trial did observe that vancomycin therapeutic drug monitoring was independently inversely associated with nephrotoxicity (adjusted OR 0.04; 95% CI 0.01–0.30) in 70 patients with hematologic malignancies. However, the generalizability of this study is somewhat limited given the patient population and routine concomitant administration of amikacin (~80%) and amphotericin B (~30%).

The 2009 version of the vancomycin consensus guidelines recommended using 30–45 mg/kg/day based on total body weight to achieve vancomycin serum trough concentrations of 15–20 mg/L [4]. The authors stated that this approach should extrapolate to an area under the curve (AUC) of ~400 mg·h/L. There were several reports of increased nephrotoxicity associated with the implementation of these guideline recommendations. The vast majority of these reports discussed the increased risk of nephrotoxicity being associated with vancomycin trough concentrations (either 15 or 20 mg/L or greater). A meta-analysis of these studies documented that a vancomycin trough of 15 mg/L or greater was associated with nephrotoxicity (OR 2.67; 95% CI 1.95–3.65) [20]. However, the authors note that nephrotoxicity was reversible in the majority of cases and that short-term dialysis was only required in 3% of nephrotoxic episodes. A case series of nine patients found obstructive tubular casts containing vancomycin in the presence of elevated serum vancomycin concentrations [52]. Eight of the nine patients had serum vancomycin concentrations of at least 35 mg/L. The authors confirmed the clinical observations by administrating vancomycin to four mice. The vancomycin-containing casts also occurred in the mice in the presence of elevated vancomycin concentrations. Vancomycin therapy should be held for at least one dosing interval if the true vancomycin trough is greater than 25 mg/L.

There was more variance in AUC with trough-based monitoring than anticipated by the original guideline authors. Neely et al. evaluated three data sets through modeling and simulation to compare obtained trough values to AUC estimations. The simulation results suggest that an AUC/MIC ≥ 400 mg·h/L can be achieved with a trough <15 mg/L in 60% of patients [53]. A retrospective study by Ghosh et al. reported that 61% of patients achieving an AUC/MIC ≥ 400 mg·h/L had a vancomycin trough <15 mg/L [54]. A prospective trial of 252 patients found that 31% of patients with an AUC/MICs ≥ 400 mg·h/L had a trough concentration <10 mg/L and 68% had a trough concentration <15 mg/L [55]. Therefore, multiple studies have shown that AUC provides a better estimate of vancomycin exposure than a single trough concentration.

A recent meta-analysis of eight observational studies (*n* = 2491) suggested a cutpoint of 650 mg·h/L for the risk of vancomycin-associated nephrotoxicity. Patients with an AUC/MIC < 650 mg·h/L were less likely to develop nephrotoxicity whether the AUC was calculated in the first 24 h period (OR 0.36; 95% CI 0.23–0.56) or second 24 h period (OR 0.45; 95% CI 0.27–0.75) [56]. Using an AUC monitoring strategy was associated with significantly lower rates of nephrotoxicity than trough-guided monitoring (OR 0.68; 95% CI 0.46–0.99). However, this finding is based on only two studies, with one retrospective study representing 90% of the total sample in the analysis.

The primary issue with all of these analyses is that they all fail to identify if increased vancomycin concentrations are the cause of nephrotoxicity or if they are increased as a result of nephrotoxicity. In addition, the reliance upon retrospective analyses and computer-generated cutpoints brings the stability of the values generated into question. The lack of randomized, controlled trials targeting different trough or AUC values is particularly concerning in that we may be continuously creating risk factors for nephrotoxicity that are never validated prospectively in a randomized trial.

### 5.2. Fluid Status

The European Society of Intensive Care Medicine issued strong recommendations (lower-level evidence) regarding the use of controlled fluid resuscitation with crystalloids and avoiding fluid overload to prevent the development of nephrotoxicity [57]. To our knowledge, no data exist assessing the association between hypovolemia and nephrotoxicity specifically related to vancomycin therapy. However, we believe continuous reassessment of volume status should take place throughout the course of treatment as part of the patient and drug monitoring process, as the detrimental effects of either hypovolemia or fluid overload have been reviewed elsewhere [58].

### 5.3. Reassessment of Nephrotoxicity Risk

We are unaware of literature that documents the clinical benefit of re-assessing the patient’s risk of nephrotoxicity. However, we feel that this should be a routine part of clinical care, as it makes common sense that assessing for the risk of adverse events should be a continual process.

## 6. Diagnosis of AKI

More than 35 definitions of acute renal dysfunction have previously been identified in the literature [59]. The most commonly utilized definitions of vancomycin-associated nephrotoxicity are consistent with the Acute Kidney Injury Network (AKIN), Kidney Disease Improving Global Outcomes (KDIGO), and Risk, Injury, Failure, Loss, End stage renal disease (RIFLE) criteria but vary between studies [60,61,62]. The 2009 version of the vancomycin consensus guidelines defines vancomycin-induced nephrotoxicity based on an increase in serum creatinine of 0.5 mg/dL or a ≥50% increase from baseline [4]. Most of these definitions allow for classification based on serum creatinine or urine output as a surrogate for the diagnosis of kidney injury. The vast majority of studies evaluating vancomycin and nephrotoxicity have focused on serum creatinine changes due to their retrospective nature. Data regarding urine output has typically not been evaluated in these retrospective evaluations due to the lack of information regarding the urine volume and/or the accuracy of the data for timing and volume charted.

Clinicians have used serum creatinine as a diagnostic criterion for AKI for decades. This surrogate measure is plagued by several issues. The accuracy of serum creatinine in estimating renal function in patients with extremes of weight (e.g., anorexics, weight lifters) or decreased muscle mass (e.g., elderly, long-term spinal cord injury patients) may be less accurate, since creatinine is a product of muscle catabolism [63,64,65]. Additionally, the kinetics of creatinine often result in a delay between kidney injury and the subsequent rise in serum creatinine. This may lead to delays in recognition and diagnosis of nephrotoxicity [66].

Various medications have also been associated with increases in serum creatinine without changes to renal function. Similar to piperacillin-tazobactam, there are several agents including trimethoprim, cimetidine, pyrimethamine, and various antiretroviral agents that have been found to increase serum creatinine through inhibition of creatinine tubular secretion without decreasing glomerular filtration rate [67,68,69,70,71].

More sensitive urinary biomarkers have been evaluated recently as potential replacement(s) to serum creatine, given its issues in estimating glomerular filtration rate and/or diagnosing AKI. These candidates to serve as next-generation biomarkers include urinary KIM-1, neutrophil gelatinase-associated lipocalin (NGAL), interleukin-18 (IL-18), cystatin C, clusterin, fatty acid binding protein-liver type (L-FABP), and osteopontin [72]. Animal studies have suggested that KIM-1 and/or clusterin monitoring may identify nephrotoxicity in the setting of vancomycin exposure more quickly [73,74]. Continuous monitoring of renal function is also being explored as an alternative to conventional methods. The optimal molecule to facilitate the continuous monitoring has not been identified in the last ten years [75]. Additional research is needed to assess the feasibility and utility of these monitoring methods in clinical practice.

## 7. Prognosis of AKI

In general, patient outcomes after AKI are poorly described in current literature. The rate of in-hospital death associated with AKI ranges from 15–60% depending upon the patient population studied and the degree of renal impairment reached [76,77]. The presence of any KDIGO stage of AKI has been associated with death up to 10 years (OR 1.30; 95% CI 1.1–1.6) from being admitted to an intensive care unit (ICU) [78]. This effect was not observed when only patients who survived the first 28 days were evaluated (OR 1.26; 95% CI 1.0–1.6).

Nephrotoxicity in the setting of vancomycin therapy appears to be reversible in most cases. Jeffres and colleagues observed that 73% of patients with nephrotoxicity had reductions in serum creatinine levels to near baseline by hospital discharge [6]. A larger study found that 81% of cases of nephrotoxicity resolved [11]. A meta-analysis found that short-term dialysis was only required in 3% (6/192) of all patients who developed nephrotoxicity [20]. None of these patients required long-term dialysis. A retrospective study evaluating the timing of serum creatinine lowering in patients with AKI observed that serum creatinine remained 50% above baseline for a median duration of seven days (interquartile range (IQR0 3, 20 days) [10]. While vancomycin had higher rates of nephrotoxicity (18.2% vs. 8.4%) compared to linezolid in a randomized, controlled trial of patients with MRSA nosocomial pneumonia, its use was not associated with 60-day mortality (26.6% vs. 28.1%) [79].

## 8. Conclusions

Vancomycin remains a first-line agent for the treatment of MRSA infections despite different generations questioning its nephrotoxic potential. The lack of prospective randomized, controlled trials evaluating various vancomycin dosing strategies and/or combination empiric therapy regimens has left clinicians to depend on data from cohorts (primarily retrospective) to evaluate vancomycin’s nephrotoxic potential. These gold standard trials could have a dramatic impact by informing which dosing strategies and vancomycin-based combinations are safest to use, particularly in patients at risk of nephrotoxicity.

Clinicians should not fear using vancomycin in the absence of these data. Patients who develop AKI while receiving vancomycin infrequently require acute renal replacement therapy and even fewer chronic therapy The short-term mortality increase associated with AKI may be an indicator of more acute illness, or it could even be a result of more aggressive/risky interventions being used in these patients. We would advise to evaluate the patient in addition to the serum creatinine instead of basing treatment decisions solely on laboratory values.

We are hopeful that the novel biomarkers for kidney injury will help clear the issues regarding the timing of renal injury and better elucidate the potential causes. Several medications can compete with creatinine via tubular secretion. This competition creates uncertainty regarding whether serum creatinine increases represent damage to the kidneys or not. The most frequent instance is piperacillin-tazobactam being prescribed along with vancomycin to provide empiric gram-negative and anaerobic coverage. Having a more accurate marker of kidney function could potentially help clinicians from unnecessarily avoiding this combination. In addition, some clinicians are choosing alternatives that may result in other safety issues in select patients (e.g., cefepime and neurotoxicity) [80]. Antimicrobial stewardship efforts can be conducted in the meantime to decrease the duration of combination empiric therapy. This approach has additional benefits outside of the AKI prevention.

We recognize that some clinicians may seek to avoid vancomycin in patients with multiple risk factors for nephrotoxicity. This makes common sense even though there are no data to validate this approach. One study that sought to evaluate the random assignment of other anti-MRSA agents versus vancomycin in patients at risk of nephrotoxicity failed to observe a difference between these approaches [81]. However, another study has shown improvements in clinical outcomes by avoiding nephrotoxins [82]. This is why we advocate using a patient-specific approach that evaluates the patient, severity of illness, and concomitant medications in order to make an informed decision that takes the specific patient’s baseline (and ongoing) risk into account.

## Figures and Tables

**Table 1:** Summary of the patient care process to assess the risk of nephrotoxicity in patients being considered for vancomycin therapy.

Stage of Patient Care Process	Characteristic	Measures	Notes
Patient assessment	Severity of illness	APACHE II Pitt bacteremia score ICU residence	Increased severity of illness has been associated with nephrotoxicity
	Concomitant disease states	Renal dysfunction	Nephrotoxicity increased whether as a cutpoint for serum creatinine or creatinine clearance. Also serum creatinine as a continuous variable.
		Increased creatinine clearance	Only found as a risk factor in one cohort to date.
	Concomitant nephrotoxins	Hypotension and/or vasopressor use	No data regarding the impact of the duration of hypotension.
		ACE inhibitor, amphotericin B, tacrolimus, loop diuretics, and tenofovir	Information regarding the impact of dose and/or duration is lacking
		Piperacillin/tazobactam	Increases diagnosis of nephrotoxicity, but may be renal-protective
Antibiotic prescription	Patient need for an antibiotic	Clinical and microbiologic assessment	Tension exists between the need for rapid adequate empiric therapy and providing antibiotics to patients with non-infectious diseases
	Patient need for vancomycin	Clinical and microbiologic assessment	Assess for risk of MRSA. Further advances in risk scores for assessing risk are needed.
Duration of therapy	Vancomycin duration	Days of vancomycin therapy	Nephrotoxicity risk increases with longer durations of therapy. Most clinical guidelines recommend seven days of vancomycin. Notable exceptions include endocarditis and osteomyelitis.
	Vancomycin discontinuation	Microbiologic assessment	Use of rapid diagnostics, nasal PCR swabs can help aid in discontinuation of vancomycin.
Monitoring	Therapeutic drug monitoring	Vancomycin concentrations	AUC goal should be 400–650 mg·h/L If a trough approach is utilized, please hold at least one dose for a trough ≥25 mg/L
	Fluid status	Intake and output reporting	Both fluid overload and hypovolemia are associated with nephrotoxicity. Accurate intake and output charting can be difficult in some practice environments.
	Reassessment of nephrotoxicity risk	See patient assessment section	

ICU: intensive care unit; ACE: angiotensin converting enzyme; MRSA: methicillin-resistant *Staphylococcus aureus*; PCR: polymerase chain reaction; AUC: area under the curve.
